# The Current Landscape of Targeted Clinical Trials in Non-WNT/Non-SHH Medulloblastoma

**DOI:** 10.3390/cancers14030679

**Published:** 2022-01-28

**Authors:** David R. Ghasemi, Gudrun Fleischhack, Till Milde, Kristian W. Pajtler

**Affiliations:** 1 Hopp Children’s Cancer Center Heidelberg (KiTZ), 69120 Heidelberg, Germany; t.milde@kitz-heidelberg.de; 2 Division of Pediatric Neurooncology, German Cancer Research Center (DKFZ) and German Consortium for Translational Cancer Research (DKTK), 69120 Heidelberg, Germany; 3 Department of Pediatric Oncology, Hematology and Immunology, Heidelberg University Hospital, 69120 Heidelberg, Germany; 4 Pediatric Oncology and Hematology, Pediatrics III, University Hospital of Essen, 45147 Essen, Germany; gudrun.fleischhack@uk-essen.de; 5 Clinical Cooperation Unit Pediatric Oncology, German Cancer Research Center (DKFZ) and German Consortium for Translational Cancer Research (DKTK), 69120 Heidelberg, Germany

**Keywords:** non-WNT/non-SHH medulloblastoma, group 3 medulloblastoma, group 4 medulloblastoma, precision medicine, molecularly guided trials

## Abstract

**Simple Summary:**

Medulloblastoma is a form of malignant brain tumor that arises predominantly in infants and young children and can be divided into different groups based on molecular markers. The group of non-WNT/non-SHH medulloblastoma includes a spectrum of heterogeneous subgroups that differ in their biological characteristics, genetic underpinnings, and clinical course of disease. Non-WNT/non-SHH medulloblastoma is currently treated with surgery, chemotherapy, and radiotherapy; however, new drugs are needed to treat patients who are not yet curable and to reduce treatment-related toxicity and side effects. We here review which new treatment options for non-WNT/non-SHH medulloblastoma are currently clinically tested. Furthermore, we illustrate the challenges that have to be overcome to reach a new therapeutic standard for non-WNT/non-SHH medulloblastoma, for instance the current lack of good preclinical models, and the necessity to conduct trials in a comparably small patient collective.

**Abstract:**

Medulloblastoma is an embryonal pediatric brain tumor and can be divided into at least four molecularly defined groups. The category non-WNT/non-SHH medulloblastoma summarizes medulloblastoma groups 3 and 4 and is characterized by considerable genetic and clinical heterogeneity. New therapeutic strategies are needed to increase survival rates and to reduce treatment-related toxicity. We performed a noncomprehensive targeted review of the current clinical trial landscape and literature to summarize innovative treatment options for non-WNT/non-SHH medulloblastoma. A multitude of new drugs is currently evaluated in trials for which non-WNT/non-SHH patients are eligible, for instance immunotherapy, kinase inhibitors, and drugs targeting the epigenome. However, the majority of these trials is not restricted to medulloblastoma and lacks molecular classification. Whereas many new molecular targets have been identified in the last decade, which are currently tested in clinical trials, several challenges remain on the way to reach a new therapeutic strategy for non-WNT/non-SHH medulloblastoma. These include the severe lack of faithful preclinical models and predictive biomarkers, the question on how to stratify patients for clinical trials, and the relative lack of studies that recruit large, homogeneous patient collectives. Innovative trial designs and international collaboration will be a key to eventually overcome these obstacles.

## 1. Introduction

Medulloblastoma (MB) is the most common malignant central nervous system (CNS) tumor of infancy as well as early childhood and accounts for a significant share of both cancer-related morbidity and mortality in this age group [1,2]. Historically, MB has been diagnosed based on histomorphological features and stratified into four respective types: Classic, Large Cell/Anaplastic, Desmoplastic/Nodular, and MB with Extensive Nodularity [3]. However, a growing body of work on the (epi-)genetic background of MB resulted in a first consensus in 2012 that established four molecular groups: WNT, SHH, Group 3, and Group 4 (Gr. 3/Gr. 4) [4]. These developments have culminated in the recognition of a molecularly defined MB classification system in the WHO classification of CNS tumors [5]. The recently published fifth edition includes the molecular MB diagnoses WNT-activated, SHH-activated/*TP53*-mutated, SHH-activated/*TP53*-wildtype, and non-WNT-/non-SHH alongside one category for histologically defined MB [6]. DNA methylation-based classification has developed into an accepted tool to confidently stratify MB into its molecular groups [7,8]. Other methods, such as nanoString-based RNA-assays and PCR-arrays, are also applied for subgrouping [9,10].

WNT MB accounts for about 10% of all patients. These tumors are defined by mutations related to the respective pathway, such as *CTNNB1* or *APC*, and are generally associated with excellent survival rates [8,10,11,12,13,14]. Notably, germline *APC* mutations, known to cause familial polyposis syndromes, also predispose to WNT MB [8,15,16]. Within the SHH group, which comprises about 25–30% of all cases, mutations and copy number variations (CNVs) of SHH-pathway members (*PTCH1, SUFU, SMO, GLI1/2, MYCN*) and alterations of *TP53* and *TERT* are characteristic genetic hallmarks [4,8,10,12,13,17]. Furthermore, this group also accounts for the majority of patients suffering from cancer predisposing germline mutations. Cancer predisposition syndromes that predispose to SHH MB include, amongst others, Li–Fraumeni syndrome and Gorlin–Goltz syndrome [8,15,16,18,19]. The prognosis of SHH MB varies strongly and ranges from favorable to dismal, for instance, depending on the presence of a *TP53*-mutation [20,21,22,23]. Both WNT- and SHH-activated MB can be further subdivided into two and four subgroups, respectively, and represent comparably well understood and clearly defined molecular groups [6,12,24].

In contrast, the situation concerning Gr. 3 and Gr. 4 MB is more complex. Together, both groups form the spectrum for non-WNT/non-SHH MB, which accounts for roughly 60% of all cases and remains the genetically most heterogeneous and least understood fraction of MB cases [25]. Gr. 3 MB shows the worst outcome across all MB groups and frequently harbors amplification or overexpression of the *MYC*-gene [10,26,27,28]. Gr. 4 MB is mostly associated with intermediate risk and is enriched for somatic alterations of genes involved in chromatin remodeling and histone modification, such as *KDM6A,* and notably *MYCN* amplification in roughly 6% of cases [8,13,29]. However, in contrast to the WNT and SHH groups, no single somatically mutated gene is present in more than 5–10% of either Gr. 3 or Gr. 4 MB patients, constituting a significant challenge in the development of innovative treatment strategies for these children [13]. Furthermore, recent molecular studies with so far unprecedented sample sizes have shown that both molecular groups do not only show significant group-specific heterogeneity, but also partly overlap in the sense that a number of cases were not unambiguously assignable to either of the two groups [13,24,30,31]. These findings are underlined by the fact that Gr. 3/4 MB share several genetic alterations, such as structural whole-chromosome abnormalities or enhancer hijacking-mediated overexpression of *GFI1* and *GFI1B*, which has been shown to drive tumor formation in vivo in combination with *MYC*-overexpression [10,32]. Furthermore, a recent single cell sequencing study reported the existence of intermediate tumors that share features of both Gr. 3 and Gr. 4 transcriptional programs in roughly 20% of all Gr. 3/4 cases [33].

A consensus study in 2019 established a subdivision of Gr. 3/4 MB into eight molecular subgroups including shared ones that differ in methylation profiles, genetic alterations, epidemiological, and clinical features [34]. The increased level of complexity concerning stratifying non-WNT/non-SHH MB is taken into account in the current 2021 version of the WHO classification of CNS tumors by recommending a layered, integrated diagnostic approach that accounts for both molecular group and subgroup alongside histological appearance [6]. Recently, a randomized clinical trial which tested an intensified therapeutic regime that included carboplatin and isotretinoin resulted in significantly improved outcomes for children suffering from Gr. 3 MB, illustrating the potential of molecular-guided patient stratification [35].

Throughout the last decade, the combined efforts of the MB community have led to an increased understanding of the biological underpinnings of this heterogeneous disease and a refined molecular classification that lays the foundation for new ways of treating MB in the future. In this noncomprehensive review, we describe the current landscape of precision-medicine trials for which non-WNT/non-SHH MB patients are eligible, with a strong focus on preclinically validated molecular targets and treatment strategies that are or have recently been explored in the clinic.

## 2. Molecular Targets and Treatment Strategies in Non-WNT/Non-SHH MB

### 2.1. Kinase Inhibitors

**Figure 1:**
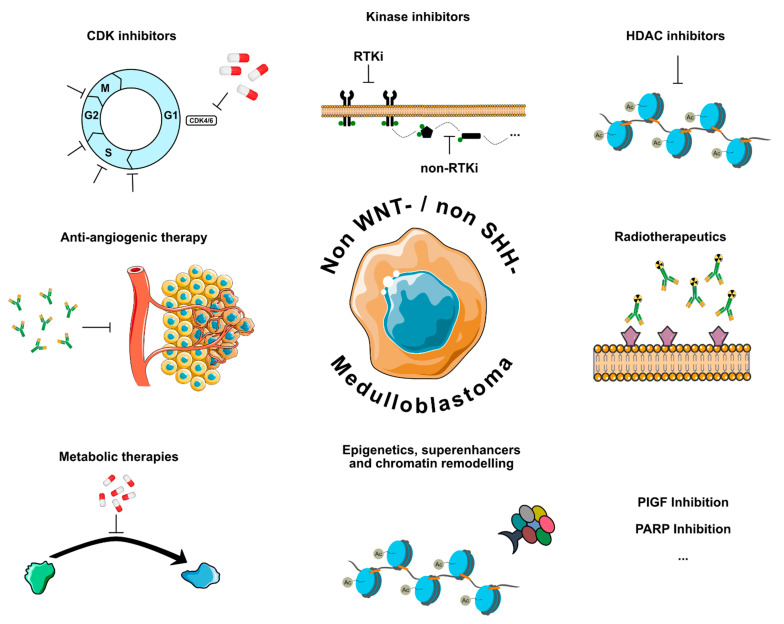
Personalized and molecular therapeutic strategies currently tested for non-WNT/non-SHH MB. RTKi = receptor tyrosine kinase inhibitor.

Kinase inhibitors have developed into one of the standard drug classes in the repertoire of personalized oncology [36,37], and a multitude of kinase inhibiting compounds has been tested preclinically in MB, most of them targeting enzymes that are involved in cell cycle regulation. However, activating mutations in receptor tyrosine kinases, which are frequently detected in other CNS malignancies such as glioblastoma, are rare in MB [8]. While cycline-dependent kinase (CDK) inhibitors are discussed separately, this paragraph gives a short overview on other kinase inhibitors that are currently tested in (non-WNT/non-SHH) MB (Figure 1). WEE1 is a tyrosine kinase that is involved in G2 checkpoint regulation and expressed in non-WNT/non-SHH MB, and the WEE1-inhibitor Adavosertib has been tested preclinically in Gr. 3/4 model systems [38,39]. In both studies, Adavosertib showed synergistic effects with the chemotherapeutic agents cisplatin and gemcitabine in suppressing MB tumor growth. This finding may be based on the fact that WEE1-inhibition is more effective in the presence of replication stress and DNA damage [40]. The clinical use of Adavosertib for pediatric cancer patients has been investigated in combination with Irinotecan; however, only one MB patient was enrolled in the respective trial (NCT02095132), which showed an acceptable safety profile with a maximum tolerable dose (MTD) of 85 mg/m^2^/day [41,42]. Importantly, studies in glioblastoma showed heterogeneous and partly limited distribution of Adavosertib across the blood–brain barrier [43,44,45,46]. Similar to WEE1, the protein kinase CHK1 is involved in cell cycle regulation and has been proposed as a potential vulnerability in Gr. 3 MB in vitro and in vivo [47,48,49]. Preclinical evidence from an in vivo Gr. 3 MB model suggests that therapeutically meaningful concentrations of Prexasertib can reach the brain [48,50]. A first-in-pediatrics trial recently illustrated that Prexasertib monotherapy was well tolerated in a pediatric mixed solid tumor population (NCT02808650/ADVL1515) using a dose of 150 mg/m^2^ administered i.v. on days 1 and 15 of a 28-day cycle, although no objective responses were reported [51]. Currently, one phase I trial investigates the use of Prexasertib in combination with established DNA-damaging agents used in medulloblastoma to evaluate tolerance and pharmacokinetics in recurrent or refractory non-WNT/non-SHH (NCT04023669) (Table 1). Due to their mechanism of action, which allows cells with high levels of DNA damage to enter the cell cycle, both Adavosertib and Prexasertib may be dependent on synergistic chemotherapy that induces high levels of genomic damage to unfold their full efficacy [39,40,47]. It should be noted that for both WEE1 and CHK1, preclinical efficacy is based on overexpression and not on somatically altered genes, with the potential downside that the mere overexpression of tyrosine kinases alone might not represent a long-lasting target in the context of personalized therapy [52].

### 2.2. CDK Inhibitors

CDKs are centrally involved in the positive regulation of cell cycle activity and their activity is frequently dysregulated in numerous forms of cancer [53,54]. The FDA and EMA approval of the three CDK4/6 inhibitors Palbociclib, Ribociclib, and Abemaciclib for the treatment of certain forms of breast cancer have also sparked great interest in the concept of CDK inhibition in other tumor types, including MB. However, potential pitfalls, for example, resistance mechanisms such as loss of function mutations in the Rb gene, need to be taken into account when testing CDK inhibitors in pediatric tumors. Amongst many other biological mechanisms, CDKs stabilize MYC-family proteins, which make CDK inhibition an interesting therapeutic option, especially for non-WNT/non-SHH MB [55]. Indeed, several CDKs are altered in a subset of non-WNT/non-SHH MB, for instance, in the form of CDK6-amplification in Gr. 4 MB [8,10,34]. Throughout the last years, preclinical evidence that CDK inhibitors could provide new therapeutic opportunities for non-WNT/non-SHH MB has grown, especially for MYC-amplified MB and in combination with other drugs, such as BET bromodomain-inhibition [56,57,58]. Several trials are currently testing CDK inhibitors for CNS tumors in children (Table 1). A recently published phase I trial determined pharmacokinetic properties and the MTD of Palbociclib monotherapy in both mild and heavily pretreated children with progressive brain tumors, including four MB patients, albeit without molecular information (NCT02255461/PBTC-042) [59]. As anticipated from trials in adults, one of the main dose-limiting side effects was neutropenia. No patient showed an objective response to Palbociclib treatment, suggesting that future trials may need to focus on combinatorial approaches. Currently, one treatment arm of the pediatric MATCH basket trial (NCT03155620) investigates the use of Palbociclib as a monotherapy (NCT03526250), whereas two industry-sponsored trials are combining CDK inhibitors Palbociclib and Abemaciclib with different chemotherapy regimens for relapsed or refractory pediatric solid tumors (NCT03709680/NCT04238819). Further, the currently running SJDAWN trial includes one study arm in which patients with recurrent/relapsed non-WNT/non-SHH MB and ependymoma will be treated with gemcitabine and Ribociclib. Results of this study may allow first estimates for duration of objective response and progression-free survival in a biologically homogeneous group of non-WNT/non-SHH MB (NCT03434262).

### 2.3. HDAC Inhibitors

Histone deacetylases (HDACs) are important epigenetic regulators of transcription and can induce gene repression via chromatin remodeling and altered transcription factor binding [60]. Throughout the last decade, a large body of work has established HDAC inhibition as a promising therapeutic avenue in MB, with a special focus on MYC-amplified non-WNT/non-SHH MB [61,62,63,64,65,66,67]. A recent study demonstrated that HDAC inhibitors stabilize the MYC protein and thus lead to reduced DNA binding and ultimately lower expression of MYC target genes [63]. Furthermore, a combined phase I/II dose escalation trial showed overall manageable toxicity and partial responses in a fraction of patients (including eight MB patients) for the HDAC inhibitor Vorinostat [68]. This is in line with another study that showed a good safety profile for the combination of Vorinostat and Temozolomide in a small cohort of children with relapsed CNS malignancies, including two MB patients (NCT01076530) [69]. Currently, several trials are testing different HDAC inhibitors and therapeutic strategies (Table 1): One early phase I trial is investigating the infusion of Panobinostat into the fourth ventricle of recurrent MB patients (NCT04315064). A second study is testing the pan-HDAC and PI3K inhibitor Fimepinostat in a cohort of pediatric brain tumor patients, including MB (NCT03893487/PNOC016). Vorinostat in combination with Isotretinoin and chemotherapy was investigated in infants with MB and other embryonal brain tumors; however, the results are not yet published (NCT00867178/PBTC-026). HDAC inhibition modifies T-cell regulation and can augment response to check-point inhibition by reducing the number of myeloid-derived suppressor cells [70,71,72,73], thereby creating an immunogenic tumor microenvironment including the induction of major histocompatibility complexes and neoantigens. Therefore, combination of HDAC inhibitors with immune checkpoint inhibitors as in the biomarker-driven INFORM2 NivEnt trial (Nivolumab and Entinostat) represents an exciting approach for non-WNT/non-SHH MB (NCT03838042) [52,74]. While none of these studies is restricted to non-WNT/non-SHH MB, it seems likely that a significant percentage of enrolled patients will be relapsed or progressive Gr. 3/4 MB patients.

### 2.4. Antiangiogenic Therapy

Antiangiogenic drugs belong to the first successes of personalized medicine and are currently used in a wide range of oncological entities and disease settings. Several studies reported that genes, such as *VEGFA* and *HIF1A*, which are important for malignant neovascularization, are expressed in MB [75,76,77]. One publication showed that as compared to other MB groups, *VEGFA*-levels are elevated in Gr. 3 MB, which corresponded to vessel density and correlated with survival [78]. These findings indicate a potential role of antiangiogenic therapy for non-WNT/non-SHH MB. Axitinib, a multikinase inhibitor that also targets neovascularization, has shown efficacy in vitro and in vivo in c-MYC amplified MB models [79,80]. Furthermore, a number of case reports and small retrospective studies have indicated that antiangiogenic drugs, such as Bevacizumab, can achieve objective response in some MB patients, especially in combination with chemotherapy [81,82,83,84,85,86,87]. A prospective, randomized phase II trial by the Children’s Oncology Group (ACNS082/NCT01217437) showed significantly reduced risk of death as the primary endpoint in children suffering from relapsed MB when treated with temozolomide/irinotecan and bevacizumab as compared to temozolomide/irinotecan alone [88]. For 36/105 patients, molecular data were reported, and 27/36 cases were assigned to the non-WNT/non-SHH group. Additional evidence for such therapeutic strategies may be derived from the metronomic MEMMAT trial that evaluates the activity of a multidrug antiangiogenic approach enrolling relapsed MB (NCT01356290) (Table 1).

### 2.5. Radiotherapeutics

MB has long been known to be sensitive to radiotherapy, and the idea to use radioactive isotopes that are conjugated to antibodies that are selectively targeting cancer cells in the CNS is more than thirty years old [89]. One phase II trial showed encouraging results and a good safety profile for the radioconjugated antibody 131I-3F8 in a MB cohort of *n* = 42, including several long-term survivors in a heavily pretreated patient collective (NCT00445965) [90]. 131I-3F8 targets GD2, a cell-surface disialoganglioside that is expressed on a variety of cancers, including MB. Unfortunately, no information on molecular diagnoses was reported, therefore the potential of 131I-3F8 for non-WNT/non-SHH MB cannot yet be judged. Furthermore, two industry-led trials testing radiotherapeutics for which MB patients are eligible are currently ongoing (Table 1): One phase I trial with the radioconjugate CLR131 for solid pediatric tumors including CNS malignancies (NCT03478462), and another phase I/II study that specifically addresses MB patients and tests the B7-H3 targeting radioconjugate 177Lu-DTPA-omburtamab. Interestingly, this study offers a cohort expansion phase for which SHH, Gr. 3 and Gr. 4 MB patients are eligible only (NCT04167618). Additionally, one study will combine Omburtamab-I131 with Irinotecan/Temozolomide and Bevacizumab for the treatment of recurrent MB and ependymoma (NCT04743661).

### 2.6. Metabolic Therapies

Compared to epigenetics, transcriptomics, and genomics, MB metabolomics is a field still in its infancy. Thus, only few therapeutic concepts exist to target the metabolism of MB in general and non-WNT/non-SHH MB specifically. One approach is the inhibition of IDO1, an enzyme that is centrally involved in tryptophane catabolism and has been identified as a key player in creating an immunosuppressive microenvironment [91]. A small study reported IDO1 expression in a cohort of 27 MB samples, including 16 non-WNT/non-SHH patients [92]. A phase I trial that used the IDO1 inhibitor Indoximod in combination with Temozolomide in a pediatric population suffering from recurrent/refractory CNS malignancies, was completed last year and enrolled 81 patients (NCT02502708). Although results from the trial are not yet published, a phase II study applying the combination of Indoximod with radiochemotherapy is currently open for participation (NCT04049669) (Table 1). Besides tryptophane, polyamine metabolism has been identified as a potential vulnerability in several cancers, including SHH MB, since polyamines are involved in the regulation of a number of cellular processes [93,94]. A possibility to interfere with polyamine synthesis is the inhibition of the enzyme ornithine decarboxylase (ODC) using the drug Difluoromethylornithine (DFMO/Eflornithine), a potent inhibitor of ODC, which has been tested for various types of cancer, including neuroblastoma [94]. DFMO is already approved for the treatment of sleeping sickness and hirsutism, making it an interesting repurposing candidate. However, its use remains debated due to toxicity concerns and pharmacokinetic challenges [94]. While preclinical studies are lacking, DFMO is currently tested in non-WNT/non-SHH MB patients in an expanded use setting (NCT03581240) and in a phase II trial exploring it as maintenance therapy for high-risk MB (NCT04696029). Taken together, as in other malignancies targeting metabolomic vulnerabilities in non-WNT/non-SHH MB may represent a valuable approach to enhance therapeutic mainstays. To date, scarce preclinical and clinical evidence does not allow for any conclusion or outlook in this molecular subgroup.

### 2.7. Epigenetic Therapies, Chromatin Remodeling, and Superenhancers

Chromatin remodeling and other epigenetic mechanisms represent key drivers in non-WNT/non-SHH MB. [8,12] A significant percentage of genomic alterations in these tumors is detected in genes that are involved in chromatin remodeling, histone modification, enhancer hijacking, and other epigenetic regulatory mechanisms, especially in Gr. 4 MB [8,10,13,32]. Thus, the underlying pathways have long been identified as potential vulnerabilities in non-WNT/non-SHH MB. However, the biological function of the involved genes and complexes, such as the SWI/SNF or PRC2 complex, is often context-specific and extremely complicated, and their roles in MB formation and proliferation are not yet fully understood [95,96,97,98,99]. For instance EZH2, the catalytic subunit of the PRC2 complex, has been reported to be overexpressed in non-WNT/non-SHH MB, and the same study showed that inhibition of EZH2 in two SHH MB cell lines led to decreased proliferation and induced apoptosis [100]. Conflicting with these results, another more recent study reported that inactivation of *EZH2* accelerated tumorigenesis in a *MYC*-driven Gr. 3 MB mouse model, illustrating the potential caveats of applying EZH2 inhibitors in the clinic [101]. Two other studies demonstrated a modest benefit in overall survival for the EZH2 inhibitor Tazemetostat in MB xenografts and antitumor effects in in vitro models of Gr. 3 MB [102,103]. Notably, there is also growing evidence from large scale diagnostic studies that overexpression of target genes in absence of other alterations is not predictive for response to a targeted treatment [52]. Tazemetostat was available for progressive or recurrent non-WNT/non-SHH MB patients with confirmed *SMARCA4*-loss of function mutations (~9% of Gr. 3 MB [8]) in a currently active, but not recruiting MATCH phase II trial (NCT03213665) (Table 1). Thus, it will be interesting to learn if respective patients were enrolled and what objective response or progression-free survival rates resulted from treatment.

Roughly 20–25% of all non-WNT/non-SHH MB harbor alterations in either *MYC* or *MYCN*. Whereas these genes have long been identified as two of the most important oncogenes, directly targeting them has proven challenging [104]. Thus, indirectly inhibiting the effect of *MYC* family genes has attracted considerable interest in oncological research. The BET/bromodomain family (BRD/BET) consists of several genes that are key players in regulating the expression of oncogenes and in the organization of superenhancers [105]. Amongst many other functions, BRD/BET bromodomains recognize histone acetylation and subsequently activate gene expression mechanisms. Targeting BRD/BET proteins interferes with *MYC*-dependent transcription and was shown to be a promising strategy in a number of *MYC(N)*-driven cancers, including MB and neuroblastoma [56,106,107,108,109,110,111,112,113]. Currently, a number of BRD/BET inhibitors are developed, with promising preclinical results and importantly also in a pediatric setting [112]. Additionally, the first pediatric phase I trial testing the BRD/BET inhibitor BMS-986158 was started in 2019 and is open for participation of pediatric cancer patients with *MYC(N)*-amplification or high copy number gain, thus potentially offering a new therapeutic option for the most aggressive form of non-WNT/non-SHH MB (NCT03936465) (Table 1).

### 2.8. Immunotherapy

#### 2.8.1. Immune Checkpoint Inhibitors

Immune checkpoint inhibitors that interfere with the PD1/PDL1- and CTLA4-mediated immunosuppressive crosstalk between malignant and immune effector cells are celebrated as one of the most important developments in oncology during the last decade [114]. However, these strategies rely on the presence of tumor-infiltrating lymphocytes and especially the blockade of the PD1/PDL1-axis is at least partly dependent on the expression of PDL1 on the respective tumor cells. A series of studies used immunohistochemistry and bioinformatic deconvolution to assess immune infiltration and PDL1-expression in MB [115,116,117,118,119,120,121,122,123]. Although most studies only analyzed small case series or cohorts of MB patients, together they suggest that MB is an immunologically “cold” tumor with only sparse immune infiltration. Furthermore, apart from one notable exception [118], all studies showed negligible or no PDL1-expression at all, especially for non-WNT/non-SHH MB [115,116,118,119,121,122]. Additionally, studies investigating intratumoral heterogeneity using single cell RNA-sequencing reported only minor infiltration of tumor-specific lymphocytes and a diverse spectrum of myeloid cells and microglia, which potentially contribute to an immunosuppressive tumor microenvironment [33,124,125]. These findings indicate that it may be challenging to implement immune checkpoint inhibition as part of future treatment strategies for these patients, at least in form of a monotherapy.

**Figure 2:**
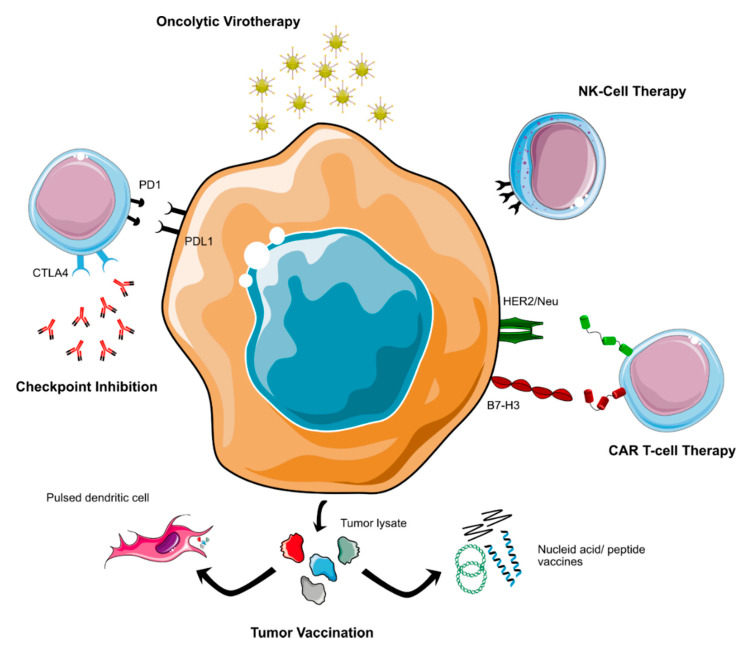
Immunotherapeutic approaches currently tested for non-WNT/non-SHH MB.

However, limited preclinical findings still warrant further validation of immune checkpoint blockade as a therapeutic concept for non-WNT/non-SHH MB (Figure 2), e.g., as part of a combination therapy that induces a “hotter” tumor microenvironment such as the abovementioned INFORM2 NivEnt trial [74,117,122]. Currently, several clinical trials that offer immune checkpoint inhibitors as a monotherapy are also open to patients with recurrent or relapsed MB (NCT02359565: Pembrolizumab and NCT03173950: Nivolumab) (Table 1). However, for the latter trial only patients >18 years of age are eligible, and these patients most often do not harbor non-WNT/non-SHH MB. Another phase II trial that tested either Nivolumab as a monotherapy or in combination with Ipilimumab is in the stage of finalization (NCT03130959). Lastly, a currently running industrial trial tests the combination of Nivolumab with the immunostimulant Bempegaldesleukin, a recombinant form of IL-2, in children and young adults with treatment-resistant cancer (NCT04730349). It should be noted though that none of these trials is exclusively recruiting non-WNT-/non-SHH or even generally MB-patients, and it remains to be seen if enough patients will be enrolled to allow for subgroup-specific analysis.

#### 2.8.2. Cellular Immunotherapy

The success of cellular immunotherapy as a new treatment for hematologic malignancies has sparked intensive research activities that aim at translating these novel therapeutic concepts into the clinic for CNS- and other solid tumors. Currently, two main concepts of cellular immunotherapy are tested in MB patients: chimeric antigen receptor (CAR) T-cells and natural killer (NK) cell therapy [126]. CAR T-cells are produced by isolating the patient’s own cytotoxic T-cells, which are then equipped with a CAR that can recognize any form of surface marker and subsequently activates the T-cell to mount an immunological attack against the target cell [127]. It is of crucial importance to choose target antigens that are highly expressed on cancer cells, but not or only negligibly on normal tissue to avoid severe on target/off tumor-toxicity. Currently, several promising targets are investigated for MB-directed CAR T-cell therapy: HER2/Neu, B7-H3 (also called CD276), EPHA2, IL-13Rα2, and PRAME [128,129,130,131,132,133]. HER2/Neu plays an important role as an oncogenic antigen in a number of solid tumors, including breast cancer and glioblastoma, and has also been identified as a possible target for MB-directed cellular therapy both in vitro and in vivo [129,130,134]. Currently, one phase I trial testing HER2/Neu-specific CAR T-cells is recruiting and open to MB patients (NCT03500991), and a recently published interim analysis of the first three enrolled patients (anaplastic astrocytoma, ependymoma) showed no dose-limiting toxicity and presented evidence of immune activation (Table 1) [135]. B7-H3/CD276 is a pancancer antigen that is strongly expressed by MB [128]. Similar to HER2/Neu, B7-H3-specific CAR T-cells have shown preclinical activity against non-WNT/non-SHH MB-models both in vitro and in vivo as well as in a number of other (pediatric) cancers, including atypical teratoid/rhabdoid tumor (AT/RT), another aggressive CNS-tumor of early childhood [128,136,137,138]. These preclinical findings provide a strong rationale to test B7-H3 targeting CAR T-cells in the clinic, which is currently undertaken in one phase I study that enrolls children with B7-H3 positive CNS-malignancies, including MB (NCT04185038). While HER2/Neu and B7-H3 targeting CARs have already been translated into the clinic, several other targets may be of interest based on preclinical data: one study showed strong expression of EPHA2 and IL-13α2 in human Gr.3 MB and subsequently tested both monovalent EPHA2- and trivalent EPHA2-/HER2/Neu-/IL-13α2-targeting CAR T-cells in a Gr. 3 MB-mouse model, with promising results [132]. An IL-13α2 directed CAR T-cell trial is recruiting adult patients with leptomeningeal metastases, including MB, and the results may be of interest to inform future trials in pediatric populations (NCT04661384). Furthermore, another study provided evidence in vitro that PRAME might represent a promising target for CAR T-cell therapy in MB [133]. Additionally, two more phase I CAR-T cell trials are currently open to pediatric patients with CNS-malignancies, including MB. However, the respective target antigens EGFR806 and GD2 have not been tested preclinically in MB patients (NCT03638167 and NCT04099797). Lastly, it should be noted that the delivery of CAR T-cells to the brain poses challenges due to the role of the blood–brain barrier, which could lower the effectiveness of intravenously applied cellular immunotherapies [129]. To date, the best application route for CAR T-cell in neurooncology has not yet been determined. However, the majority (4/5) of currently running CAR T-cell trials for which MB patients are eligible will use intraventricular/intracavital cell delivery, which circumvents the blood–brain barrier.

An exciting alternative to CAR T-cells is the use of NK cells that may offer certain advantages in comparison, such as lower side effects and increased resistance to immune evasion strategies of the tumor [139]. Several preclinical studies have shown that in principle, NK cells are able to recognize and eliminate MB cells; however, additional stimulation may be needed to arrive at clinically meaningful cytotoxic activity levels [140,141,142,143,144]. Interestingly, one study showed a higher and more consistent sensitivity to NK cells in vitro for non-WNT/non-SHH as compared to SHH MB cell lines [141]. In contrast, another study reported significantly higher expression levels of CD1d, an antigen recognized by NK cells, on SHH as compared to Gr. 4 MB [142]. Clearly, further studies are needed to arrive at a conclusive answer concerning which MB groups are the most promising target for NK cell therapy. The safety and feasibility of NK cell therapy for pediatric brain cancer has recently been shown by a phase I trial that also enrolled five MB patients, although without reporting molecular diagnoses (NCT02271711) [145].

#### 2.8.3. Tumor Vaccinations

Cancer vaccine approaches harness the ability of off-the-shelf or patient-specific antigens to induce an antitumoral immune reaction [146]. Several different strategies have been developed, for instance using antigen pulsed dendritic cells, peptides, and nucleic acid vectors. Tumor vaccines have been studied for a long time; however, several early phase trials have recently shown great potential in adult glioblastoma patients [147,148,149]. One study tested dendritic cells that were pulsed with tumor lysate-derived antigens. As compared to AT/RT and high grade glioma, the five included MB patients showed only modest therapy response, albeit no molecular information is available [150]. The results of another phase I trial that used RNA-pulsed dendritic cells and also enrolled patients with recurrent MB and glioma are pending (NCT03615404). An additional trial applies another strategy that uses a peptide-based approach (NCT03299309). Lastly, MB patients are currently also eligible for a first-in-pediatrics trial that investigates a long peptide vaccine that targets the apoptosis inhibitor survivin (NCT04978727) (Table 1). Similar to most of the treatment strategies presented so far, none of these trials is restricted to non-WNT/non-SHH MB. Furthermore, so far, no definitive conclusions can be drawn from the reported data concerning differences in the efficacy of vaccination therapies for MB molecular subgroups.

#### 2.8.4. Other Immunotherapeutic Approaches

Apart from checkpoint inhibition, increasingly more immunomodulatory approaches are entering the clinical stage. Two studies for a mixed pediatric population with CNS malignancies are currently testing immunostimulatory agents (Table 1): firstly, the antibody APX005M that targets CD40 (NCT03389802) and secondly the immune modulator WP1066, which inhibits the transcription factor STAT3 and therefore interferes with the JAK2/STAT3-pathway (NCT04334863). Additionally, oncolytic viruses, which have recently shown promising results for pediatric high grade glioma, have been proposed as a therapeutic option for MB [151,152]. Preclinical studies suggest that Gr. 3 MB might be a potential candidate for oncolytic virus therapy, and several different viral vectors have been tested throughout the last decade [153,154,155,156,157,158,159,160,161]. Oncolytic virus studies that are currently recruiting MB patients include the investigation of a modified measles vaccine (NCT02962167), Herpes Simplex Virus (NCT03911388), and one open phase IB trial that will assess possible agonistic effects between mesenchymal allogenic cells and an adenovirus-based virotherapy (NCT04758533) (Table 1). Two additional early phase studies that are open to MB patients are currently active, but not recruiting; these are testing reovirus in combination with Sagramostim and a modified poliovirus (NCT02444546, NCT03043391).

### 2.9. Other Molecular Therapeutic Approaches

In addition to the treatment approaches discussed thus far, several other concepts should be mentioned briefly (Table 1). Firstly, the inhibition of placental growth factor (PGF) has been proposed as a promising strategy across all MB subgroups and was tested in a phase I trial using the monoclonal antibody TB-403 (NCT02748135) [162]. The results have been presented at the AACR annual meeting 2021 and were encouraging both in terms of safety and efficacy according to the producing company; however, a peer-reviewed publication is not available to date [163]. Another interesting treatment approach represents the inhibition of DNA damage response pathways using poly (ADP-ribose) polymerase (PARP) inhibitors, a relatively new drug class that has received numerous approvals for breast and ovarian cancer patients with germline BRCA1/2 mutations in the last years [164]. This could be especially relevant to a subset Gr. 3/4 MB, since these are the molecular groups that are enriched for (germline) BRCA2- and PALB2-mutations [13,16]. Thus, it will be interesting to see whether non-WNT/non-SHH patients will be included in currently running trials using PARP inhibitors (NCT03233204 and NCT04236414). Lastly, several preclinical studies with different compounds indicate that the concept of sensitizing cancer cells to radiotherapy using small molecules could be potentially interesting for non-WNT/non-SHH MB [165,166,167]

## 3. Discussion

In this review, we aimed at summarizing the current landscape of targeted clinical trials and most important molecular targets for non-WNT/non-SHH MB. Although a multitude of promising, molecularly informed new treatment options is tested to date, several significant challenges remain:

(i) Preclinical in vitro and in vivo models for non-WNT/non-SHH MB are almost entirely restricted to MYC-driven Gr. 3 MB and, thus, do not reflect the true heterogeneity of these malignancies. This is especially true for Gr. 4 MB, for which faithful models are still broadly lacking despite it being the most frequent form of MB [8,134,168].

(ii) The considerable heterogeneity of genetic driver events in non-WNT/non-SHH MB makes it more challenging to systematically test therapeutic approaches in reasonably sized patient cohorts. Although there was significant progress in the last years, the biological underpinnings of non-WNT/non-SHH MB are not yet fully understood. Thus far, no targetable alteration has been identified that would be present in more than a small share of non-WNT/non-SHH MB cases. Additionally, many theoretically targetable alterations are not predictive of response [52].

(iii) The rise of molecular diagnostics has further subdivided MB. Whereas these developments offer exciting chances to arrive at more homogeneous, biologically defined risk groups, they also pose a considerable challenge to clinical research, since it becomes increasingly more difficult to collect a significantly large number of patients for clinical trials. Additionally, different competing stratification strategies are conceivable. For instance, non-WNT/non-SHH MB could be stratified into eight molecular subgroups based on DNA methylation patterns. However, this approach would collide with dividing patients according to genetic alterations, such as *MYC*-amplification, which is not restricted to just one of the abovementioned molecular subgroups [34]. Furthermore, the number of available patients will be too small to test several drug candidates for the same molecular target by different companies, making a prioritization of most promising drug candidates based upon evaluated and consensus criteria necessary [169,170]. Again, this already challenging situation is complicated by the fact that any monotherapy is likely to be of only modest success, and combinatorial approaches will probably have to be tested to arrive at clinically meaningful results. These could include combinations between conventional chemotherapeutic and targeted agents, as currently tested by the MEMMAT (NCT01356290) or SJDAWN-trials (NCT03434262). However, therapy regimens consisting of several immunotherapeutic or targeted drugs, such as the INFORM2 NivENT study, also hold promise to arrive at new options to treat children suffering from non-WNT/non-SHH MB [74]. Recently, an international consensus statement defined the minimum preclinical testing requirements that should be met before translating a drug into the early phase clinical setting [169]. Notably, while these recommendations strongly advocate for the use of several in vivo models (PDX, genetically engineered mice models, etc.) for any new compound before advancing to a phase I trial, this kind of solid proof-of-concept evidence is broadly missing in the field of non-WNT/non-SHH MB. As highlighted earlier, this issue is largely rooted in the lack of faithful preclinical models and has thus been to a certain extent unavoidable in the past. However, as soon as more and better in vivo models are available, any given drug (or combination of drugs) should be tested rigorously in a standardized set of preclinical models before entering the clinic, as pointed out by Vassal et al. [169].

(iv) Throughout the last decade, the vast majority of phase I/II trials in which MB patients were enrolled did not report molecular information; thus, for a large share of the studies conducted so far that tested new treatment modalities for MB, information on the efficacy in non-WNT/non-SHH MB is lacking.

(v) Due to the rarity of pediatric cancer in general, most of the past and currently open trials are not restricted to MB, let alone non-WNT/non-SHH MB. The small number of non-WNT/non-SHH MB patients per trial will be a considerable challenge to generate the level of high-quality evidence that is necessary to change the current therapeutic standard. Conducting classic randomized phase III therapy optimization trials for MB will become increasingly more challenging, especially when it comes to recurrent disease. The basis to overcome these challenges lies in extensive international cooperation and a shared strategy between academia and industry. A blueprint on how research activities can be coordinated and hastened to benefit non-WNT/non-SHH MB patients represents the international ACCELERATE platform, which, for instance, recently published a roadmap for the development of BRT/BET inhibitors [112]. Further efforts are necessary to standardize preclinical models and to determine criteria to choose the most promising amongst several drug candidates with the same target as this, for example, is realized by the pediatric preclinical proof-of-concept platform ITCC-P4 (https://www.itccp4.eu, accessed on 12 December 2021).

Innovative and creative solutions to arrive at solid evidence levels for new drugs will be of paramount importance. New trial designs, for instance, based on Bayesian statistics, “pick the winner” concepts or in the form of molecular basket and umbrella trials, promise to overcome these issues eventually; however, they also pose methodological challenges [171]. Several comprehensive pediatric precision oncology programs such as pediatric MATCH, MOSCATO-01, the ZERO Childhood Cancer Program, and the INFORM platform (Individualized Therapy for Relapsed Malignancies in Childhood) have been established over the recent years to allow for assignment of patients to matching trials [52,172,173,174,175]. These registries could serve as a tool to stratify non-WNT/non-SHH MB patients into molecular-guided early phase I clinical trials. These will be necessary even in the presence of new drugs that are developed solely for non-WNT/non-SHH MB, since the large heterogeneity within this subgroup will probably prevent the design of a randomized trial in which all non-WNT/non-SHH MB patients can be included. In order to extract as much information as possible from every single case, conducting molecular diagnostics for patients who were enrolled on past trials should be pursued wherever archived material is still available for retrospective testing. Additionally, every future trial should comprehensively assess molecular characteristics of non-WNT/non-SHH MB to determine the role of biomarkers and to identify the most promising drug combinations for given targets. Although a multitude of innovative treatment modalities is theoretically available for non-WNT/non-SHH MB already and even newer concepts are currently developed, such as refined ways of targeting epigenetic alterations in cancer or strategies to directly target MYC-family genes [104], none of these concepts currently are standing out.

## 4. Conclusions

Non-WNT/non-SHH MB belongs to the most heterogeneous and complex forms of embryonal brain tumors in children. Throughout the last decade, our understanding of its biological underpinning has grown considerably, and these findings are slowly translating from bench to bedside. Whereas a multitude of therapeutic options, ranging from kinase inhibitors to immunotherapy and strategies to target the epigenome, is available to non-WNT/non-SHH MB patients in theory, the vast majority of these trials is still in phase I and not restricted to this patient group. A refined preclinical pipeline with new model systems, coordinated international efforts, and innovative trial and research strategies will be necessary to arrive at new therapeutic options and eventually improved clinical results for non-WNT/non-SHH MB.

## Figures and Tables

**Table 1:** Active trials with molecular targets relevant to non-WNT/non-SHH MB. CTx = chemotherapy, RTx = radiotherapy.

Drug Class	Molecular Target	Drug	Trial Number
Kinase inhibitors	WEE1	Adavosertib/CTx	NCT02095132
	CHK1	Prexasertib/CTx	NCT04023669
CDK inhibitors	CDK4/6	Palbociclib	NCT03526250
	CDK4/6	Palbociclib/CTx	NCT03709680
	CDK4/6	Abemaciclib/CTx	NCT04238819
	CDK4/6	Ribociclib/CTx	NCT03434262
HDAC inhibitors	pan-HDAC	Panobinostat	NCT04315064
	pan-HDAC/PI3K	Fimepinostat	NCT03893487
	HDAC class I,II,IV	Vorinostat/CTx	NCT00867178
	HDAC class I,III/PD1	Entinostat/Nivolumab	NCT03838042
Anti-angiogenic therapy	VEGF-A	Bevacizumab/CTx	NCT01356290
Radiotherapeutics	-	CLR131	NCT03478462
	B7-H3	177Lu-DTPA-omburtamab	NCT04167618
	B7-H3/VEGF-A	Omburtamab-I131/Bevacizumab/CTx	NCT04743661
Metabolic therapy	IDO1	Indoximod/CTx/RTx	NCT04049669
	ODC	DFMO	NCT03581240
	ODC	DFMO	NCT04696029
Epigenetic therapy	EZH2	Tazemetostat	NCT03213665
	BRD/BET	BMS-986158	NCT03936465
Immune checkpoint Inhibition	PD1	Pembrolizumab	NCT02359565
	PD1	Nivolumab	NCT03173950
	PD1/CTLA4	Nivolumab/Ipilimumab	NCT03130959
	PD1/CD122	Nivolumab/Bempegaldesleukin	NCT04730349
Cellular immunotherapy	HER2/Neu	CAR T-cells	NCT03500991
	B7-H3	CAR T-cells	NCT04185038
	IL-13α2	CAR T-cells	NCT04661384
	EGFR806	CAR T-cells	NCT03638167
	GD2	CAR T-cells	NCT04099797
Tumor vaccinations	-	PEP-CMV	NCT03299309
	Survivin	SurVaxM	NCT04978727
Immune modulators	CD40	APX005M	NCT03389802
	STAT3	WP1066	NCT04334863
Oncolytic viruses	-	Modified measles virus	NCT02962167
	-	HSV G207/RTx	NCT03911388
	-	AloCELYVIR	NCT04758533
	-	Reovirus/GM-CSF	NCT02444546
	-	PVSRIPO	NCT03043391
Other approaches	PGF	TB-403	NCT02748135
	PARP	Olaparib	NCT03233204
	PARP	Olaparib	NCT04236414
